# Energy Efficient Sparse Connectivity from Imbalanced Synaptic Plasticity Rules

**DOI:** 10.1371/journal.pcbi.1004265

**Published:** 2015-06-05

**Authors:** João Sacramento, Andreas Wichert, Mark C. W. van Rossum

**Affiliations:** 1 INESC-ID & Instituto Superior Técnico, Universidade de Lisboa, Porto Salvo, Portugal; 2 Institute for Adaptive and Neural Computation, School of Informatics, University of Edinburgh, Edinburgh, United Kingdom; University College London, UNITED KINGDOM

## Abstract

It is believed that energy efficiency is an important constraint in brain evolution. As synaptic transmission dominates energy consumption, energy can be saved by ensuring that only a few synapses are active. It is therefore likely that the formation of sparse codes and sparse connectivity are fundamental objectives of synaptic plasticity. In this work we study how sparse connectivity can result from a synaptic learning rule of excitatory synapses. Information is maximised when potentiation and depression are balanced according to the mean presynaptic activity level and the resulting fraction of zero-weight synapses is around 50%. However, an imbalance towards depression increases the fraction of zero-weight synapses without significantly affecting performance. We show that imbalanced plasticity corresponds to imposing a regularising constraint on the *L*_1_-norm of the synaptic weight vector, a procedure that is well-known to induce sparseness. Imbalanced plasticity is biophysically plausible and leads to more efficient synaptic configurations than a previously suggested approach that prunes synapses after learning. Our framework gives a novel interpretation to the high fraction of silent synapses found in brain regions like the cerebellum.

## Author Summary

Recent estimates point out that a large part of the energetic budget of the mammalian cortex is spent in transmitting signals between neurons across synapses. Despite this, studies of learning and memory do not usually take energy efficiency into account. In this work we address the canonical computational problem of storing memories with synaptic plasticity. However, instead of optimising solely for information capacity, we search for energy efficient solutions. This implies that the number of functional synapses needs to be small (sparse connectivity) while maintaining high information. We suggest imbalanced plasticity, a learning regime where net depression is stronger than potentiation, as a simple and plausible means to learn more efficient neural circuits. Our framework gives a novel interpretation to the high fraction of silent synapses found in brain regions like the cerebellum.

## Introduction

The brain is not only a very powerful device, but it also has remarkable energy efficiency compared to computers [1]. It has been estimated that most of the energy used by the brain is associated to synaptic transmission [2]. Therefore to minimise energy consumption, the number of active synapses should be as low as possible while maintaining computational power [1, 3, 4]. The number of active synapses is the product of the activity and the number of synapses. Energy can thus be reduced in two ways: 1) by employing a *sparse neural code*, in which only few neurons are active at any time, 2) by removing synapses leading to *sparse connectivity*, leaving only few synapses out of many potential ones. This latter process is also called dilution of the connectivity. Remarkably, during human development brain metabolism neatly tracks synapse density, rapidly increasing after birth followed by a reduction into adolescence (e.g. compare the data in [5] to [6]).

Most computational algorithms of learning, however, optimise storage capacity without taking energy efficiency into account (but see [3]) and as a result only limited agreement between models and experimental data can be expected. The best studied artificial example of learning is the perceptron which learns to classify two sets of input patterns. Despite its simplicity, results of perceptron learning are crucial as they for instance guide the design of recurrent attractor networks [7–9]. Provided the task can be learned, the perceptron learning rule is guaranteed to find the correct synaptic weights. The traditional perceptron learning algorithm assumes that weights can have any value and can change sign. In that case a perceptron with *N* synapses can on average learn 2*N* random patterns. At the maximum load the corresponding weight distribution is Gaussian, i.e., the connectivity is dense and hence energy inefficient [10]. If one restricts the synapses to be excitatory, the capacity is halved [9, 11].

In this work we ask which learning algorithm maximises energy efficient storage, and thus maximises the number of silent synapses while still being able to perform a learning task [3]. However, finding the weight configuration with the fewest possible (non-zero) synapses is a combinatorial *L*_0_-norm minimisation task. This is in general a NP-hard problem [12, 13] and thus difficult to solve exactly. Using the replica method from statistical mechanics it is possible to calculate limits on the achievable memory performance with a fixed number of synapses [10], but such methods do not yield insight on how to accomplish this. An earlier approach prunes the smallest synapses after learning. If synapses are to be removed after learning, this procedure is optimal [14, 15]. Yet, as we will show it is far better to incorporate a sparse connectivity objective during the learning process.

Here we explore imbalanced plasticity as a simple and biologically plausible way to reduce the number of required synapses and thus improve information storage efficiency. In many memory models the amount of potentiation and depression are precisely matched to the statistics of the neural activity [16–19], but here we deliberately perturb the optimal plasticity rule by introducing a bias towards depression. This imbalanced plasticity finds weight configurations that require less functional synapses and that are thus more energy efficient.

## Results

### The model

We consider a recognition task from positive examples [20–22]. The perceptron should learn to give a response whenever a sample from a given category is presented. In contrast to the standard perceptron algorithm, which ‘unlearns patterns’ for which the neuron should not be active, the synapses are not modified for negative samples. It has been argued that this setup is relevant to biology in particular when the set of negative samples is very large and/or its statistics unknown [22]. For instance, one might want to train a neuron to recognise fruits, but not update the synapses for all other objects. This setup is also relevant when studying reinforcement learning, where learning is gated by reward feedback elicited by positive samples. Finally, it resembles the one-class support vector machine used in statistical learning, which detects whether a sample belongs to a class and which has applications in anomaly detection [23, 24].

**Fig 1:**
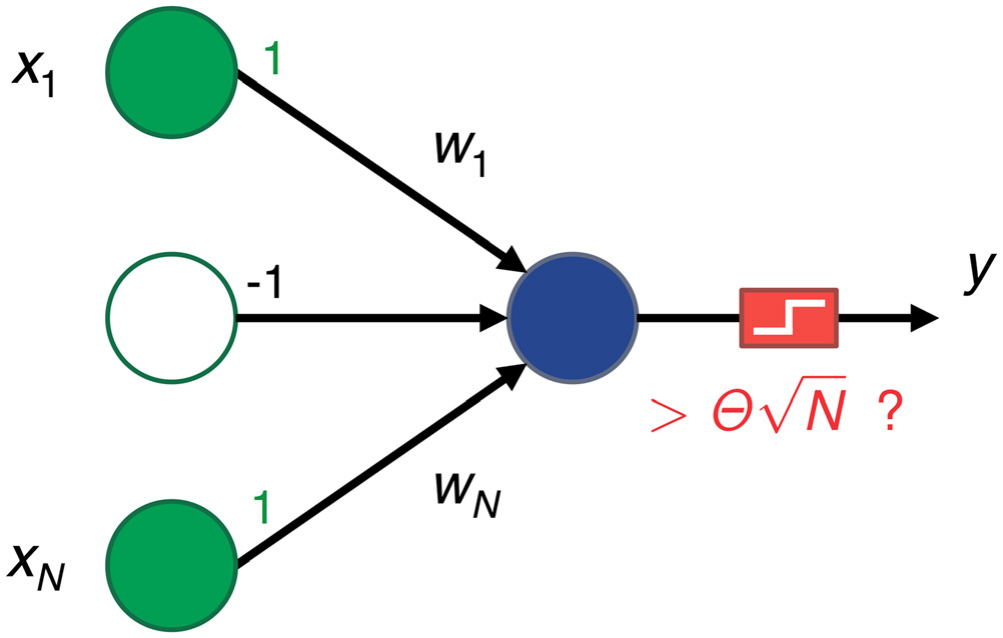
Diagram of our single neuron setup. A group of *N* input presynaptic neurons are connected to a single postsynaptic neuron. The input activity can be low, *x*_*i*_ = −1, or high, *x*_*i*_ = 1. The postsynaptic neuron performs a weighted sum of the inputs and fires whenever that sum is larger than a threshold $\theta \sqrt{N}$, otherwise it remains quiet. Each synapse *w*_*i*_ is adjusted as a function of the input activity so that the neuron remembers a set of previously seen patterns. Ideally, only these patterns should trigger the neuron; all other patterns should not.

The setup is illustrated in Fig 1. A single postsynaptic neuron calculates the weighted sum of its *N* excitatory inputs and compares it to a positive threshold $\theta \sqrt{N}$. Whenever $h=\sum_{i=1}^{N}w_{i}x_{i}-\theta \sqrt{N}$ is non-negative, the perceptron fires. The inputs *x*_*i*_ are randomly chosen to be -1 or +1 with equal probability, and independently of the other inputs (see below for extensions). The $\sqrt{N}$ in the threshold is a mathematical convenience that ensures scaling of the system as the number of inputs is varied [11, 25].

During learning the neuron is provided with a set of *K* positive patterns, **x** ^1^, …, **x** ^*k*^, …, **x** ^*K*^. As in the standard perceptron, we cycle through the set of patterns until the task is learned. The goal of the perceptron is to ‘fire’ for all these patterns. This should be contrasted to setups in which samples are presented only once (one-shot learning), which generally lead to a lower capacity [25]. We assume that initially all weights *w*_*i*_ are zero (*tabula rasa*). The learning rule is as follows: whenever a positive pattern is presented and only if it does not lead to postsynaptic activity, the synapse is updated. For high inputs, i.e., *x*_*i*_ = 1, potentiation occurs

$$\begin{array}{c} \Delta w_{i}^{+}=a\left[1-\Theta \left(h\right)\right], \end{array}$$ (1)

where Θ(⋅) is the Heaviside step function which is zero if its argument is negative and one otherwise, and *a* ≪ 1 is the potentiation rate. Similarly, when an input *x*_*i*_ is low, the synapse depresses

$$\begin{array}{c} \Delta w_{i}^{-}=-b\left[1-\Theta \left(h\right)\right], \end{array}$$ (2)

where *b* is the amount of depression. Depression is followed by rectification so that all synapses remain excitatory, *w*_*i*_ ≥ 0 [26–30]. If the pattern does already lead to firing of the perceptron, no synapse is altered. This stop-learning condition is also present in a standard perceptron; possible biophysical mechanisms are discussed in [31].

For the simple, random pattern statistics used here, the non-negativity constraint limits the maximal amount of patterns that can be learned to *K*_max_ = *N* [9, 11], which is half of the number of patterns an unconstrained perceptron can learn. Below this limit the learning process finishes with high probability in a number of steps that is polynomial in *N*. We define the memory load *α* = *K*/*N*, which becomes *α*_max_ = 1 at the maximal load in the balanced case.

### Imbalancing plasticity promotes sparseness

Unlike the traditional perceptron rule, we allow for distinct amounts of potentiation and depression. By introducing imbalance in favour of depression the learning dynamics is biased towards the hard bound of the weight at zero. We rewrite the plasticity rule using the learning rate *ε* ≡ (*a*+*b*)/2 and an imbalance parameter *λ* ≡ (*b*−*a*)/2*ε*. Provided the synapse does not hit the zero bound, the weight update is

$$\begin{array}{c} \Delta w_{i}=\epsilon \left[1-\Theta \left(h\right)\right]\left(x_{i}-\lambda \right). \end{array}$$ (3)

The parameter *λ* is zero for balanced learning; depression is stronger than potentiation if 0 < *λ* ≤ 1. We find somewhat improved faster learning when we also depress even when the pattern has already been learned, i.e.

$$\begin{array}{c} \Delta w_{i}=\epsilon \left\{\left[1-\Theta \left(h\right)\right]\left(x_{i}-\lambda \right)-\Theta \left(h\right)\lambda \right\}. \end{array}$$ (4)

For that case it can be shown that the learning dynamics minimises the energy function

$$\begin{array}{c} E=\sum_{k=1}^{K}{\left[\theta \sqrt{N}-\sum_{i=1}^{N}w_{i}x_{i}^{k}\right]}_{+}+\lambda \sum_{i=1}^{N}w_{i}, \end{array}$$ (5)

where [⋅]_+_ denotes rectification. The first term of the energy sums over all patterns and promotes low false negative rates; it is zero if the perceptron fires, while it attributes a cost proportional to the distance to the firing threshold whenever a pattern is not yet learned. The second term acts as a linear regulariser; the depression-potentiation imbalance *λ* penalises synaptic weight configurations that have large linear norms $|\text{w}|\equiv \sum_{i=1}^{N}w_{i}$. The regularisation term has a simple interpretation, as it is proportional to the mean synaptic weight, ∣**w**∣ = *N*⟨*w*⟩. The plasticity rule, Eq 4, minimises this energy by performing a stochastic sub-gradient descent [32], projected onto the subspace {**w**: *w*_*i*_ ≥ 0, *i* = 1, …, *N*}.

Rewriting the learning rule as the minimisation of the energy Eq (5) shows explicitly why introducing imbalance towards depression promotes weight sparseness. In linear regression and classification, optimising over regularised energy functions that penalise the *L*_1_-norm $||\text{w}|{|}_{1}\equiv \sum_{i=1}^{N}|w_{i}|$ of the weights is well-known to induce sparseness [33–35]. Below the critical load *α*_max_ the weight configuration with minimal linear norm is known to be sparse [27]. Thus, the learning rule Eq (4) with imbalance *λ* > 0 will try to find solutions that satisfy the learning conditions but that are sparser than those obtained when *λ* = 0.

While the linear norm constraint promotes sparseness, it is not guaranteed to produce the sparsest possible solution. The true optimisation problem would be to minimise the *L*_0_-pseudo-norm ∣∣**w**∣∣_0_. The *L*_0_-pseudo-norm simply counts the number of non-zero synapses. However, this leads to a difficult NP-hard combinatorial optimisation task [12, 13]. Instead, optimising under the *L*_1_-norm constraint is a convex relaxation of the original problem for which efficient computer algorithms exist (e.g. [36]). Moreover, imbalancing plasticity has the advantage of being an online procedure that only requires tuning the potentiation and depression event sizes and is thus biologically plausible.

### Information and efficiency

Ideally our perceptron learns all examples, and minimises the false positive rate. To characterise the performance we present the perceptron with learned samples and lures (other random patterns), both presented with equal probability. The mutual information between the class of the input pattern and the perceptron’s output on a given trial is

$$\begin{array}{c} I=\sum_{x\in \{p,l\}}\sum_{r=0,1}\;P\left(x\right)P\left(r|x\right)\log_{2}\frac{P(r|x)}{P(r)}, \end{array}$$ (6)

where P(*x*) = 1/2 is the probability that the test pattern is a positive pattern (*p*) or negative lure pattern (*l*), P(*r*) is the probability that the perceptron remains silent or fires, and P(*r*∣*x*) is the conditional probability that we observe a given response given the true pattern class.

The information can be expressed in terms of the false positive rate *p*_01_ and the false negative rate *p*_10_. Below the critical capacity (*α* ≤ *α*_max_), the positive samples are recognised perfectly after learning, i.e. there are no false negatives (*p*_10_ = 0), so that the information is determined by the false positive rate only. As we have 2*K* trials, the total information normalised per synapse, $C=\frac{2K}{N}I$, equals

$$\begin{array}{c} C=\frac{2K}{N}(1-\frac{1}{2}[\left(1+p_{01}\right)\log_{2}\left(1+p_{01}\right)-p_{01}\log_{2}p_{01}]). \end{array}$$ (7)

Although this type of information calculation is common, we note that testing with equiprobable lures and learned patterns is somewhat sub-optimal in terms of information [37]. For the one-class perceptron, testing exhaustively with all 2^*N*^−*K* possible lures gives about 60.6% more information when *p*_01_ = 1/2 with a weak dependence on *p*_01_.

As the mutual information does not take energy efficiency into account, we consider a recently suggested capacity measure that includes the sparseness of the final weight configuration [3]. The *memory efficiency* *S* measures the information per non-zero synapse by normalising the information to the fraction of non-zero synapses *F*,

$$\begin{array}{c} S=\frac{C}{F}. \end{array}$$ (8)

Memory efficiency is thus measured in *bits per functional synapse*. Learning rules that achieve high information *C* using few resources will have high efficiency. If one assumes that a non-zero synapse has a certain energy cost (independent of synaptic weight) and a zero synapse has none, the memory efficiency *S* measures the energy cost of the stored memory.

### Imbalanced plasticity improves memory efficiency

A variant of the sign-constrained perceptron convergence theorem (see Methods) shows that the learning algorithm Eq 3 converges below a critical imbalance *λ*_max_(*α*) that depends on the memory load *α*. In computer simulations we focus on the two extreme cases, i.e., balanced (*λ* = 0) and maximally-imbalanced *λ* = *λ*_max_(*α*) plasticity. In principle it is possible to find the maximum imbalance by trying various values of *λ* and checking convergence of the learning process. However, it is much quicker to use that the problem is equivalent to learn the patterns while minimising the linear norm ∣**w**∣, see Eq 5. This was done with a linear programming solver (see Methods) which requires no manual search for the maximal imbalance.

**Fig 2:**
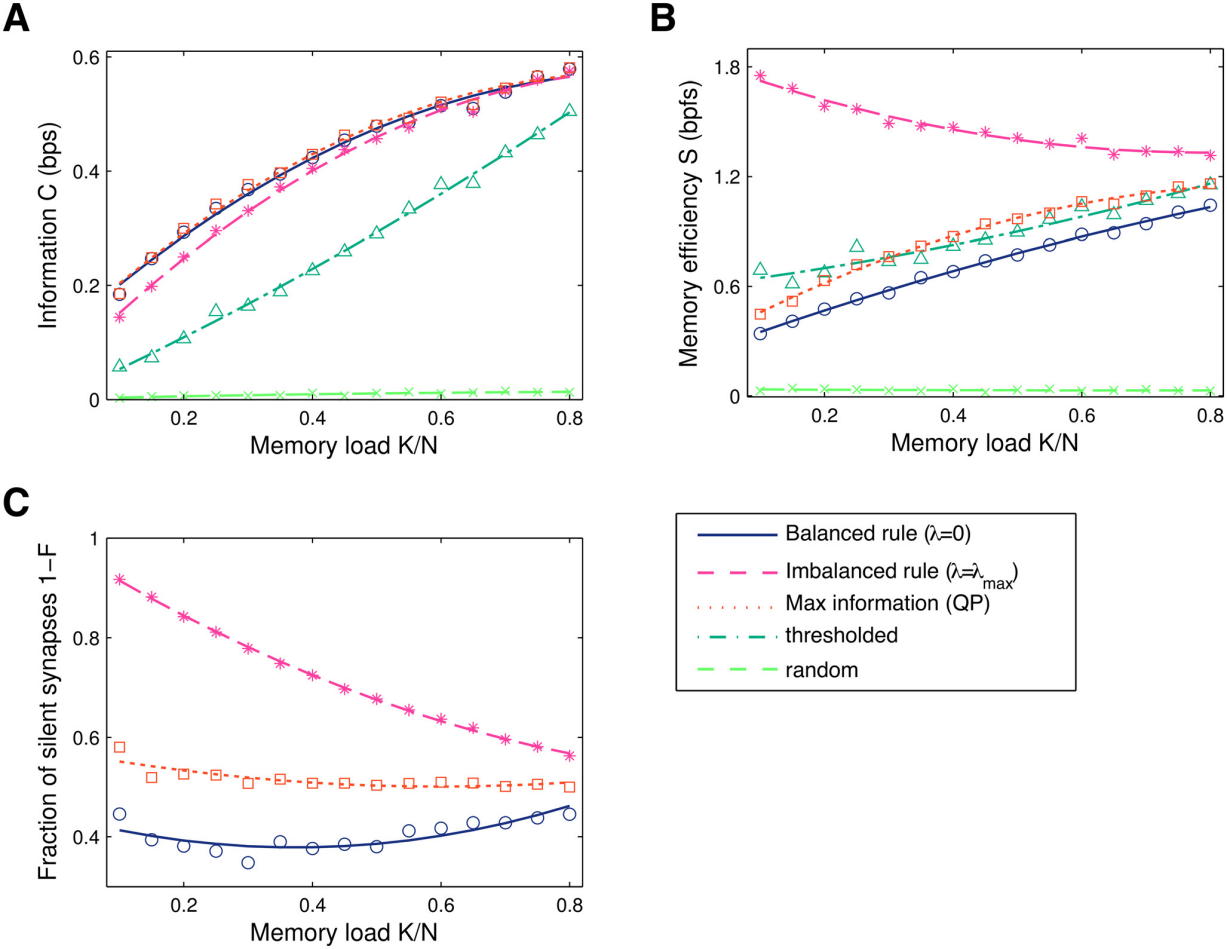
Information *C* in bits per synapse (bps), memory efficiency *S* in bits per functional synapse (bpfs) and the fraction of silent synapses 1−*F* as a function of the memory load *α* = *K*/*N*. Results from a simulation with *N* = 1000 synapses. Shown are: balanced learning where depression equals potentiation (*λ* = 0); maximal imbalance learning; the maximal-information solution found with offline quadratic programming (QP); minimal-value synapse deletion, where all weights below some threshold are set to zero; and random pruning. The two latter rules were set to delete the same number of synapses as imbalanced learning. The results for online learning were obtained under a large threshold (*θ* = 1, learning rate *ε* = 1/*N*) to maximise information (see Methods). **A**. Information. Imbalanced plasticity leads to a small information decrease and significantly outperforms thresholded pruning. Random deletion performs very poorly. Truly maximising information (QP) gives only a slight improvement in performance. **B**. Memory efficiency (information per non-zero synapse). In particular at low *α*, the imbalanced perceptron finds sparser weight configurations, boosting the memory efficiency. The curves converge as the critical loading *α* = 1 is approached. The maximal information solution (QP) is more efficient than balanced learning, but still inferior to imbalanced learning. **C**. The fraction of silent synapses. Balanced online learning (*λ* = 0) under a large threshold always leads to the appearance of silent synapses, due to the imposed hard bound at zero together with the large firing threshold. Imbalanced plasticity significantly increases sparseness, especially at lower memory loads. QP learning leads to a few more zero-weight synapses compared to balanced learning, the fraction of which remains close to 50% irrespectively of the memory load.

For strongest depression (*λ* = *λ*_max_), the information *C* is only slightly below the information of balanced learning, Fig 2A (magenta vs. blue curve). However, imbalanced plasticity provides a large increase in memory efficiency *S*, Fig 2B. The reason is that the learning dynamics converges to synaptic configurations with a considerably larger number of silent synapses, Fig 2C. As the memory load *α* increases, the efficiency approaches that of the balanced solution. This is expected; by increasing the task difficulty we are imposing additional constraints on the synaptic weights. As a result the volume of the solution space shrinks and the constraint on the mean weight has to be relieved, therefore leading to smaller gains in memory efficiency. As *α* approaches its critical value, the space of solutions collapses to a single point, i.e., no additional constraints can be imposed at critical capacity and *λ*_max_ = 0 [7].

We also considered alternative learning algorithms: first, a minimal-value pruning rule, where all weights below a certain threshold are set to zero after learning has converged. We set the deletion threshold of the offline pruning algorithm to produce the same number of zero-weight synapses as the imbalanced solution. This is optimal in the one-shot learning case [14, 15]. In this case we find a more pronounced loss of information and, interestingly, almost no efficiency increase (dark green curve). The superiority of imbalancing makes intuitive sense: imbalanced plasticity is an online protocol that accommodates for sparseness constraints by redistributing weights dynamically, while the pruning procedure is performed after learning and does not allow for further re-adjustments. Finally, we also tried random pruning after learning, which as expected, performs very poorly (light green curve).

For completeness, we compared these results to the solution that maximises information without requiring sparseness. The optimisation can be formulated as a quadratic programming (QP) problem (see Methods), and the best solution can be found with a high performance barrier method convex optimiser [38]. This algorithm clearly lacks biological plausibility, and does not provide a significant improvement in information over balanced (*λ* = 0) online learning, Fig 2A. In other words, perceptron learning works well for our problem, provided that the firing threshold *θ* is large enough (see Methods). Under QP the fraction of silent synapses slightly increases to around 50%, Fig 2C, which leads to a moderate improvement in memory efficiency, Fig 2B. Finally, one can resort to the min-over learning rule, which only applies a weight update for the pattern that evokes the minimal output *h* [39]. The synaptic weights are guaranteed to asymptotically converge (as *θ* → ∞) to the QP solution and unsurprisingly the information matches that which is obtained with the quadratic solver. This procedure is difficult to reconcile with biology as well, as each single learning iteration requires access to every pattern.

### Synaptic weight distributions

The learning algorithm and the threshold setting also determine the shape of the synaptic weight distribution. This distribution is of importance, as it can be compared to experimental data. For instance, the electro-physiologically determined synaptic weight distribution was used to link Purkinje cell learning to perceptron learning theory [28, 40]. We recorded the obtained synaptic weight histograms (see Methods), averaged over many trials (each with different pattern sets). While collecting results across trials is strictly only approximates the synaptic weight density, it is a good estimate of the actual observed distribution for a single realisation of the system, since the underlying weight density is strongly self-averaging [27, 28].

**Fig 3:**
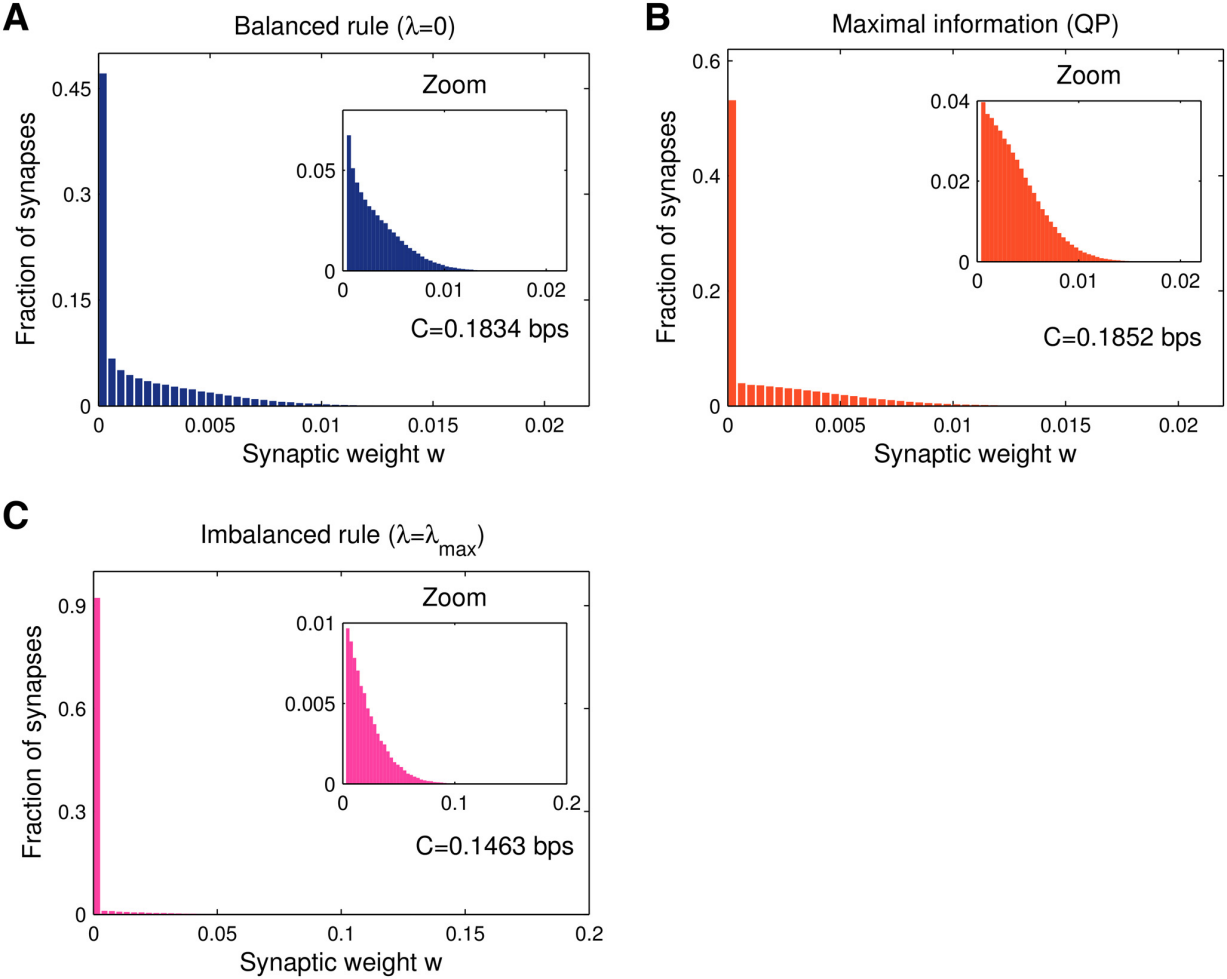
Synaptic weight histograms, information and memory efficiency at low memory load (*α* = 0.1). Data obtained averaging over a thousand simulations (*N* = 1000). **A**. For balanced learning the distribution is stretched due to the optimised learning (large threshold choice *θ* = 1 under a small learning rate *ε* = 1/*N*). As with the non-negative perceptron classifier [28], a large number of zero synapses appear. **B**. Maximal-information solution obtained via quadratic programming, with the objective set at minimising the Euclidean norm ∣∣**w**∣∣_2_. The quadratic objective function leads to a hemi-Gaussian weight distribution, again with a large fraction of silent synapses arising from the non-negativity constraint. **C**. Minimal linear norm solution (largest imbalance). As the learning task is ‘easy’ (low *α*), strong depression leads to a highly sparse synaptic configuration.

Balanced learning (*λ* = 0) leads to an approximately exponential distribution, Fig 3A. Interestingly, although the QP solution did not increase information compared to online balanced learning (Fig 2A), the shape of the distribution of synaptic weights changes considerably (cf. Fig 3A and 3B). At any memory load *α* ≤ *α*_max_ the fraction of zero-weight synapses always remains close to 50% while the remaining weights assume a truncated Gaussian distribution centred around *w* = 0. The problem that we are dealing with is thus not ‘intrinsically sparse’ in weight space. This should be contrasted with the non-negative perce ptron classifier with 0/1-coded inputs that was recently studied [28–30]. In that case, maximising information in the presence of postsynaptic noise automatically leads to sparse weight configurations (*F* < 0.5), provided that the memory load is below the critical point. Interestingly, at the critical load, the distribution becomes identical to the truncated Gaussian that we report here as the optimal one.

Imbalanced plasticity boosts the fraction of zero-weight synapses and stretches the weight distribution, Fig 3C. Although the mean weight is lower due to the increased sparseness of the weight configuration, the surviving synapses are stronger. This can be understood through theoretical arguments (see Methods). It can be shown that learning rules that lead to a large minimum postsynaptic sum, ${\text{min}}_{k}\sum_{i=1}^{N}w_{i}x_{i}^{k}$ (together with a normalisation condition that fixes the Euclidean norm ∣∣**w**∣∣_2_) give better recognition performance against lures. As some synapses are zeroed-out, specific strengthening keeps the postsynaptic sum large for learned patterns.

The non-zero weight distribution for maximal imbalance can be reasonably fitted to a compressed exponential *P*(*w*) ∼ exp(−*cw* ^*β*^), with an exponent *β* = 1.4. The two-class perceptron model yields *β* = 2 (a truncated Gaussian) at critical capacity [28]. The best fit of this type of distribution to the cerebellar data published [40] has an exponent *β* = 0.7±0.4, however it should be noted that the limited amount of data allows for a broad range of possible *β*.

### Homeostatic excitability regulation and sparse codes

Next we explore if our findings depend on the details of the coding. So far we assumed the inputs were -1 or +1, as in earlier studies of the non-negative perceptron [9, 26, 27]. This is hard to imagine biologically, unless an inhibitory partner neuron is introduced [19, 31, 41, 42]. An arguably more faithful biological model is obtained by representing low inputs as silent, *x*_*i*_ = 0 [16, 19, 20, 28, 43]. Furthermore, we wish to generalise to a case where the probability for a high input is variable rather than fixed to 1/2.

The capacity of the above model can be fully recovered without drastically changing the neural circuit. In fact, two ingredients suffice: one has to rebalance the plasticity rules as a function of the activity level *f*, and, secondly, introduce a dynamic mechanism that adapts the firing threshold as a function of the linear norm ∣**w**∣. With these modifications, both the information *C* and the memory efficiency *S* are exactly identical to those reported in the previous section.

First, we generalise the model to deal with an arbitrary coding level *f*. When *f* = 1/2, the original model is recovered up to scale factors. To preserve the zero mean, we consider activity patterns that are coded as *z*_*i*_ ∈ {−*f*,1−*f*}, with P(*z*_*i*_ = 1−*f*) = *f*. Stochastic sub-gradient descent dynamics over the energy Eq (5) gives the adjusted potentiation rule for high inputs

$$\begin{array}{c} \Delta w_{i}^{+}=\epsilon \left\{\left(1-f-\lambda \right)\left[1-\Theta \left(h\right)\right]-\lambda \Theta \left(h\right)\right\}, \end{array}$$ (9)

while depression at low inputs becomes

$$\begin{array}{c} \Delta w_{i}^{-}=\epsilon \left\{-\left(f+\lambda \right)\left[1-\Theta \left(h\right)\right]-\lambda \Theta \left(h\right)\right\}, \end{array}$$ (10)

followed by rectification. Here $h=\sum_{i=1}^{N}w_{i}z_{i}-\theta \sqrt{fN}$.

Next, a zero-mean input *z*_*i*_ is related to 0/1 coding by the simple relation *x*_*i*_ = *z*_*i*_+*f*, *x*_*i*_ ∈ {0,1}. Therefore the net input of the neuron in response to a 0/1 pattern can be written through a change of variables as

$$\begin{array}{c} h=\sum_{i=1}^{N}w_{i}x_{i}-f\sum_{i=1}^{N}w_{i}-\theta \sqrt{fN}=\sum_{i=1}^{N}w_{i}x_{i}-\gamma , \end{array}$$ (11)

where we defined a new threshold variable

$$\gamma =f\sum_{i=1}^{N}w_{i}+\theta \sqrt{fN}.$$

**Fig 4:**
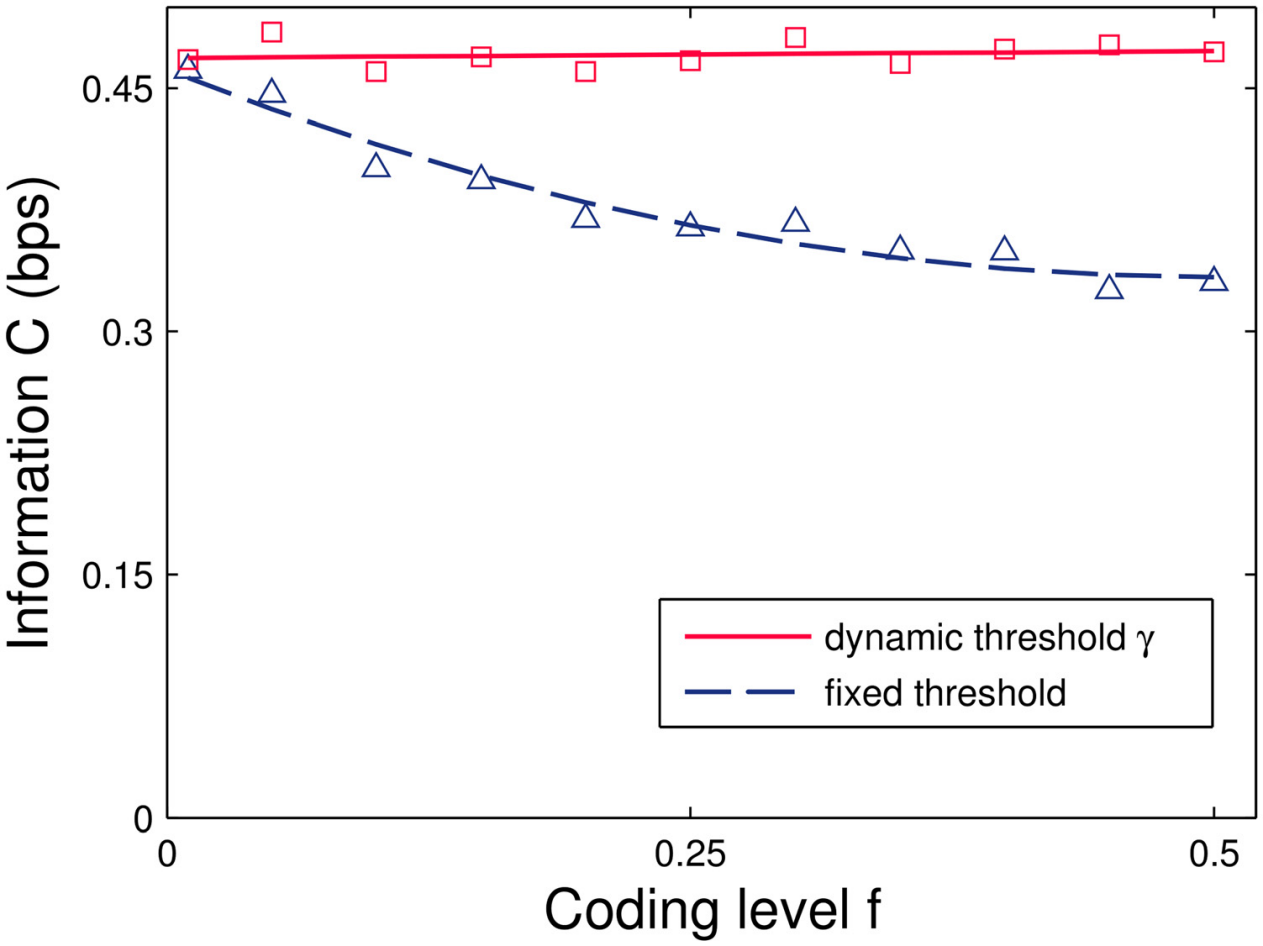
Information *C* in bits per synapse for binary (0 or 1) input patterns as a function of the input coding level *f*. Average values for the dynamic-threshold model, where *h* is given by Eq 11, and average values obtained with a fixed threshold *θfN* — note the threshold scaling with *fN* instead of $\sqrt{fN}$ due to the 0/1 input activity. Potentiation and depression were balanced (Eqs 9 and 10) to match the coding level. While the adjusted model is insensitive to *f*, the information achieved by the uncorrected model approaches that of the original one for sparse input patterns. Simulations performed at moderate memory load *α* = 0.5 and system size *N* = 1000.

Note that this threshold grows during learning so as to compensate the increasing weights. This can be viewed as a kind of homeostatic adaptation process: as learning progresses, the neuron self-regulates so that it becomes harder to reach the firing threshold. While the incorporation of an auxiliary feed-forward inhibition circuit has been used in related models to increase capacity in the presence of non-negativity constraints [19, 31, 41, 42], the mechanism here does not directly depend on the precise pattern **x** of the presented input. It thereby obviates the need for coordinated plasticity with a partner interneuron as well as for precise temporal integration of inhibitory signals. Instead it could be implemented sub-cellularly without the aid of additional circuitry. Using the adaptive threshold, the information becomes independent of the input coding level *f* (Fig 4 solid line), while it decreases when the threshold is fixed (dashed curve). We note that, unlike for two-class learning, for one-class learning a high threshold suffices to implement a large-margin classifier.

An alternative route to recover capacity is to employ sparse coding, a finding that has been previously reported for the non-negative perceptron in a more general classification framework [43]. Here the asymptotic situation is rather simple, because as *f* → 0 and *N* → ∞ the original model is recovered and performance at low *f* approaches the ideal performance, Fig 4.

### Input correlations

Activity correlations can severely limit the performance of learning rules, depending on the task and the nature of the correlations. For instance, in supervised memory tasks, Hebbian learning deteriorates under almost any type of correlation in the patterns [25, 44]. In contrast, more powerful plasticity rules equipped with a stop-learning condition, like the perceptron rule, are resistant to spatial input correlations [45], and can in some cases take advantage of input-output redundancies to store more patterns [29, 46].

**Fig 5:**
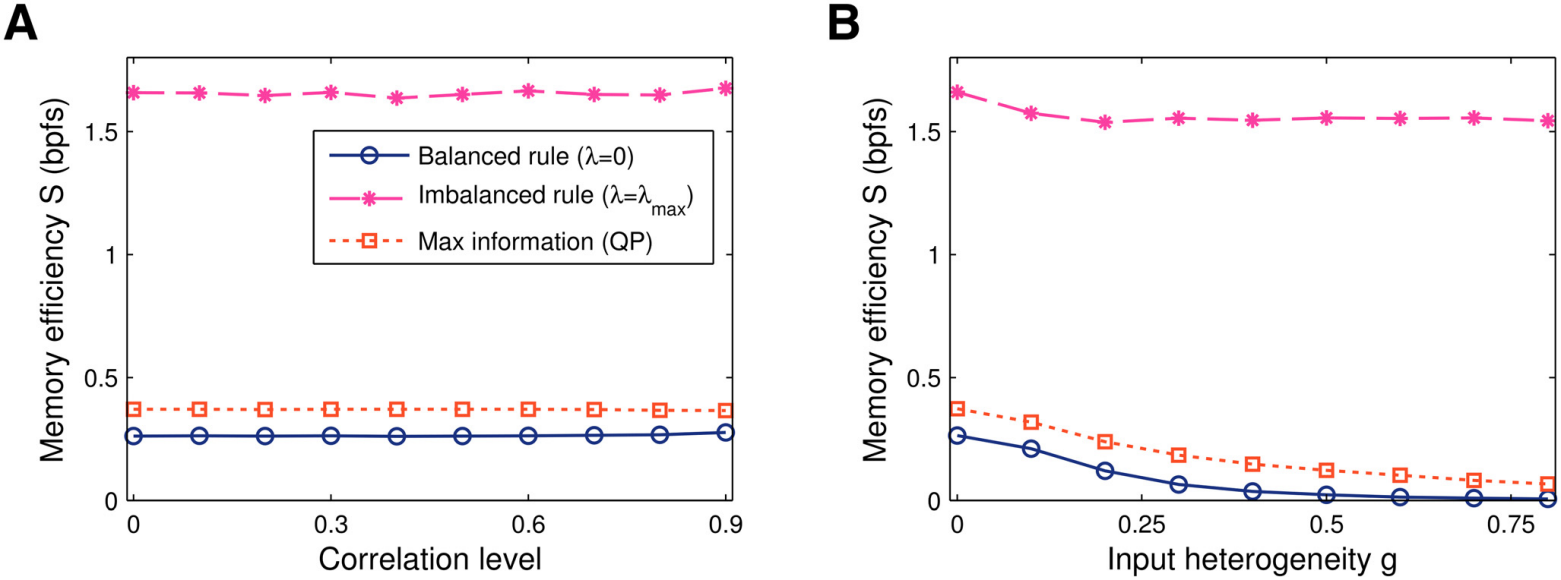
Memory efficiency vs input correlations. **A**. In case the mean input remains homogeneous, the three learning algorithms considered — balanced (*λ* = 0), maximally-imbalanced (*λ* = *λ*_max_) and maximal-information (QP) — are unaffected by spatial presynaptic activity correlations. **B**. In case of heterogenous inputs, the balanced rule (*λ* = 0) and the QP algorithm deteriorate. Imbalanced plasticity performs well, however, as it regularises the high-activity synapses while ignoring the remaining ones. As a result the memory efficiency of the maximally-imbalanced solution is approximately constant. Data obtained by averaging a hundred independent simulations at *α* = 0.1, *f* = 1/2, and *N* = 1000 synapses.

To test the robustness of imbalanced plasticity to correlated activity we draw random patterns from a generative model that induces spatial presynaptic activity correlations (characterised by a parameter *g*, see Methods, [21, 45]). We first correlated the patterns such that the mean activity remained homogeneous across the inputs. Consistent with the standard two-class perceptron without synaptic sign-constraints [45], neither the imbalanced learning, nor the balanced rule are affected by input correlation, Fig 5A.

Next, we implemented a variation of the generative model that introduces heterogeneities in the input activity rates where some inputs tend to be active more often than others. Interestingly the imbalanced rule is robust to this type of correlation, Fig 5B. Whereas the efficiency of the other rules drops off, the efficiency of the imbalanced rule remains constant. The intuitive explanation is that the high activity synapses effectively experience balanced net potentiation and depression for non-zero imbalance *λ*. The imbalanced rule finds a high-information solution by silencing and ignoring the low activity inputs and subjecting the remaining synapses to the usual imbalanced learning protocol.

### Robustness to noise

So far we have considered the recall of noise-free patterns, however, in the light of the many noise sources in the nervous system, it is important to confirm the noise robustness of the results.

First, we introduce transmission failures and spontaneous presynaptic activity, and test the learning with corrupted patterns, denoted $\tilde{\text{x}}$. An active input is switched off with probability $\delta_{10}=\text{P}({\tilde{x}}_{i}\;=\;0|x_{i}\;=\;1)$, while an otherwise silent presynaptic input fires with probability $\delta_{01}=\text{P}({\tilde{x}}_{i}\;=\;1|x_{i}\;=\;0)$. The lures are generated with a matching mean activity, ⟨*x*⟩ = (1−*f*)*δ*_01_+*f*(1−*δ*_10_), to ensure that lure statistics match the patterns.

**Fig 6:**
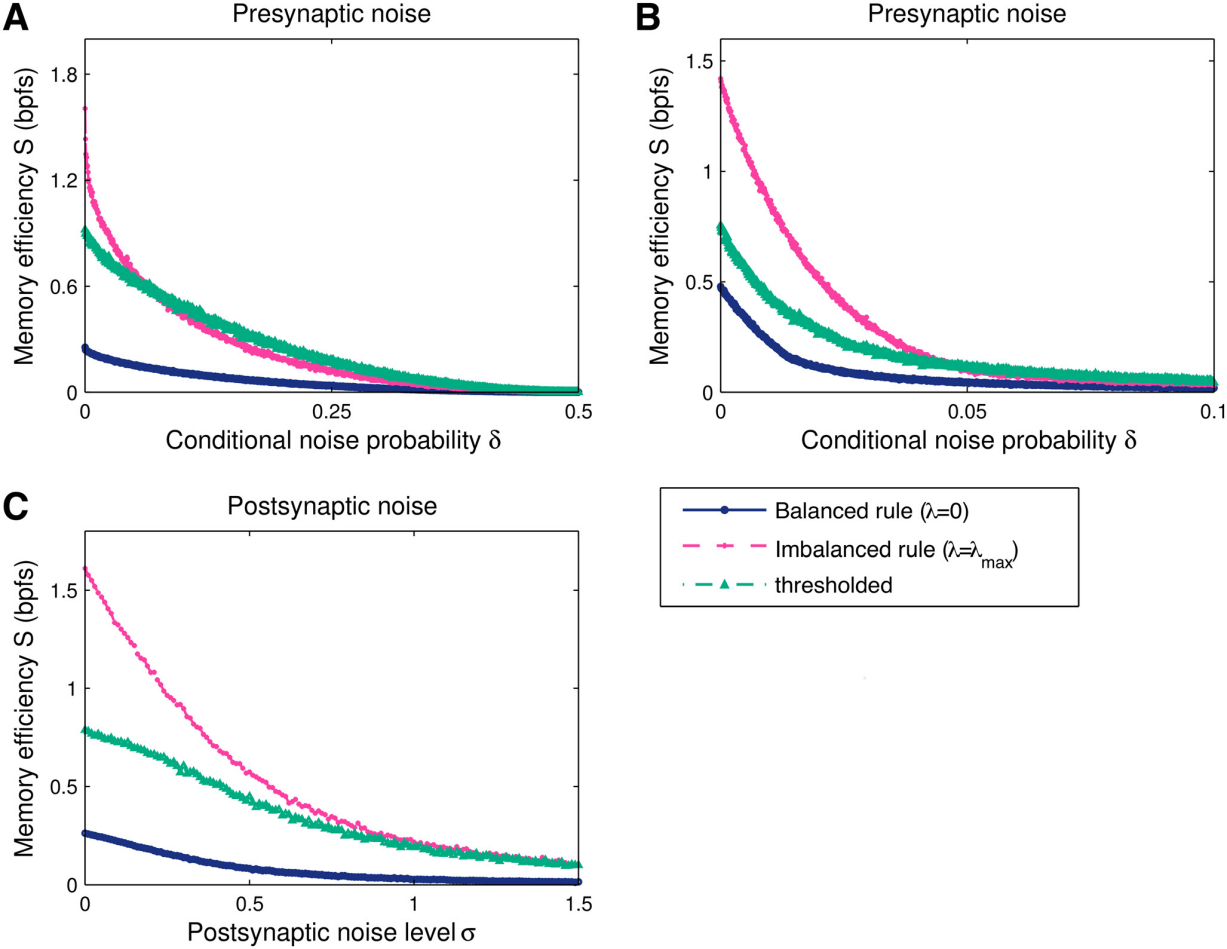
Information *C* and memory efficiency *S* versus noise level. The three solutions — balanced (*λ* = 0) and maximally-imbalanced (*λ* = *λ*_max_) plasticity, and thresholded synaptic pruning — were obtained once for a single set of *K* = 0.1*N* positive patterns (*N* = 1000 synapses) and then tested against a large number 100*K* of distorted learned patterns and lures, generated for each noise level. The firing threshold of each solution is numerically optimised to maximise information. The presynaptic noise level varied under the setting *δ*_01_ = *δ*_10_ = *δ* (see main text for details). The scale of the postsynaptic noise standard deviation was set by normalising the weights to give a unit size mean response to learned patterns. **A**. For dense patterns, *f* = 1/2, the falloff in information is steeper for imbalanced plasticity than thresholded deletion. The two solutions remain more efficient than balanced learning for all noise levels. **B**. For sparse input patterns, *f* = 0.01, the balanced solution also suffers and as long as the information is not practically zero, both the maximally-imbalanced and the thresholded pruning rules are more efficient than the balanced one. **C**. Results for a postsynaptic noise model, where the current *h* is perturbed with an additive zero-mean Gaussian random variable with standard deviation *σ*. As the postsynaptic noise does not depend on the actual learned weights, imbalanced and balanced plasticity show similar noise robustness profiles.

We examined the performance of the balanced and maximally-imbalanced rules, as well as thresholded synaptic pruning, under this stochastic synapse model, Fig 6A and 6B. The information of all three rules decreases smoothly as the input distortion increases. For dense patterns, *f* = 1/2, the efficiency of the maximally-imbalanced rule is initially the most affected by the introduction of noise, and becomes comparable to the thresholded deletion one for higher noise levels. For sparse patterns, Fig 6B, the efficiency is affected similarly by the noise for all three rules. The maximally-imbalanced and the thresholded solutions remain more efficient than balanced plasticity.

Next, we examined the role of postsynaptic current noise by adding a zero-mean Gaussian variable to the postsynaptic current *h* [28], the variance of which sets the noise intensity, Fig 6C. In contrast to the above, the magnitude of the random contributions is decoupled from the actual learned weights. For this noise model, the relative information reduction is comparable for both balanced and imbalanced plasticity.

### Tuning of the imbalance parameter

**Fig 7:**
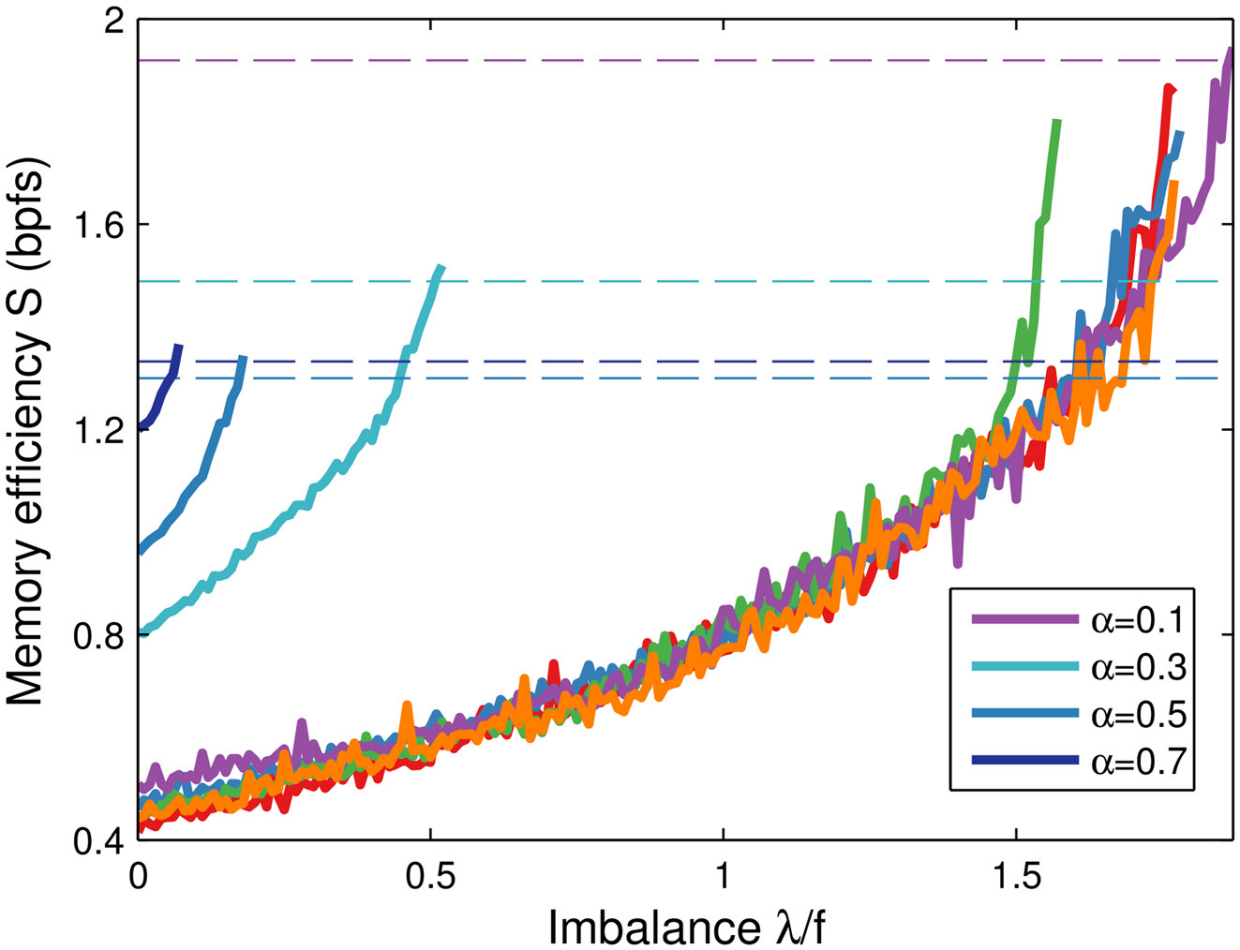
Memory efficiency *S* increases with imbalance *λ*. Efficiency is a function of imbalance for a given set of patterns. The curve stops when the learning dynamics no longer converges. Dashed horizontal lines indicate the corresponding efficiency values achieved by the linear programming solver (see Methods). The results for five independent runs at *α* = 0.1 (rightmost curves) are very similar, although finite-size effects are visible as the number of inputs is not particularly large. As predicted, the critical imbalance *λ*_max_ decreases with the memory load *α*. The learning rule only updated the synapses for patterns that did not yet lead to firing activity, Eq 3. Simulations of a neuron with *N* = 1000 synapses and coding level *f* = 0.01.

In the above the imbalance parameter *λ* was optimised for automatically in an unbiological fashion. To examine suboptimal values we simulated learning while raising *λ* towards the critical imbalance *λ*_max_, above which the learning algorithm no longer converges. The memory task difficulty, set by the memory load *α*, limits the allowed imbalance (see Methods). Indeed, we find that *λ*_max_ shrinks as *α* increases, Fig 7. Akin to the margin parameter which sets the noise robustness of the non-negative perceptron [28, 29], the actual *λ*_max_ depends on the exact set of patterns the neuron should learn. However, for random patterns drawn from the same distribution, the system is self-averaging as *N* → ∞ [7]. In simulations we observe a similar behaviour across different runs, although some finite-size effects are still apparent in networks of moderate dimension, Fig 7 (rightmost curves). In other words, *λ*_max_ can be reasonably estimated independent of the precise pattern set. Finally note that the figure implies that the parameter can be set conservatively, based on the maximum number of patterns to be expected. Of course, the efficiency gain is not maximised in this case, but still better than the balanced case.

## Discussion

The brain’s energy consumption is thought to be dominated by synaptic transmission [2, 47, 48]. We have considered how synaptic learning rules can lead to sparse connectivity and thus to energy efficient computation. We studied a one-class perceptron problem in which a neuron learns from positive examples only. One-class learning is relevant for learning paradigms such as recognition and reinforcement learning. One-class learning is also well-known in machine learning [24, 49, 50]. The two-class perceptron requires sampling the space of ‘negative’ patterns that is necessarily large under a sparse firing constraint [22] and secondly, it requires reversing plasticity (‘unlearning’) whenever appropriate. For instance, it is unclear how can a pattern be actively unlearned under spike-timing-dependent plasticity [51]. In contrast to two-class perceptrons, negative samples in the one-class perceptron do not cause plasticity which leads to further energy saving as plasticity itself is an energetically costly process [52].

We imbalance potentiation and depression to achieve sparse connectivity. In other memory tasks, the information loss can be substantial for imbalanced plasticity; for instance, postsynaptic-independent (i.e., without a stop-learning mechanism) online learning rules are severely affected when depression does not match potentiation [17–19]. However, here imbalance leads to a substantial energy reduction in storage as long as the task is below maximal capacity. Furthermore, it is robust against noise and correlated patterns. Imbalanced plasticity is not only a local and biophysically plausible mechanism, but it is also theoretically well-grounded, as it implements *L*_1_-norm regularisation, which is well-known to induce sparseness [27, 33, 34, 53]. Due to the biased drift towards zero in the learning rule, the probability of finding silent synapses is increased. Our learning rule reaches high information using a novel, biologically-plausible adaptive threshold without the need for an inhibitory partner neuron. The learning rule is unlike a previous approach to achieve sparse connectivity in which a pruning procedure removes the weakest synapses after learning [14, 15]. Such strategy can lead to as much weight sparseness as desired, but a significant drop in information and efficiency occurs.

Despite the large efficiency gain found, it should be noted that imbalanced plasticity probably does not maximise the efficiency fully. In the limit of many synapses the replica technique from statistical mechanics can provide an estimate on the minimal number of synapses required for a given performance. Extrapolation of such an analysis of the traditional perceptron without sign constraints [10], suggests that even more efficient solutions exist, although it is unclear how to obtain them via online learning. Unfortunately, the weight configuration that truly maximises memory efficiency requires resorting to an impractical and unbiological exhaustive search method, with a search time exponential in the number of synapses. A feasible alternative is to use greedy *L*_0_-norm minimisation methods [54], that are in general not guaranteed to achieve the theoretical limiting weight sparseness. Preliminary simulations suggest that the efficiency in this case is not substantially higher than when minimising the linear norm, as the increased number of zero-weight synapses is offset by a steep loss in information.

We note that sparse network connectivity can arise even when energy efficiency is not explicitly optimised for. Weight sparseness also emerges when maximising the information output of a sign-constrained classifier that is required to operate in the presence of postsynaptic noise [28, 30]. The reported weight distribution displays a large fraction of silent synapses [28]. In that learning setup, depression occurs for negative examples to drive the postsynaptic potential well below threshold and thus ensures that the activity of the neuron is suppressed even if noise is present.

In order to implement imbalanced learning various ingredients are needed. 1) As in the classical perceptron a stop-learning condition needs to be implemented. While in the cerebellum the complex spike might fulfil this role, neuromodulatory systems have also been suggested [31]. 2) The balance parameter needs to be precisely set to obtain the most efficient solution and its value depends on the task to be learned. A conservative imbalance setting will increase efficiency, but not as much. We note that the need for precisely tuned parameters is common in this type of studies, just like the standard perceptron requires a precise balance between potentiation and depression, which is also not trivially achieved biologically. 3) For one-class learning, plasticity only occurs when the neural output should be high but it is not (which contrasts the model in [28], where plasticity only occurs when the input is high). A separate supervisory input to the neuron could achieve this. Nevertheless, despite the details of this particular study the general imbalancing principle could well carry over to other systems. In particular including precise spike-timing perceptron learning [55, 56], or temporal STDP [57]. In the latter case, interestingly, energy constraints have also been used to define unsupervised learning rules.

Our study is agnostic about the precise mechanism of pruning. There is a number of biophysical ways a synapse can be inactivated [58, 59]: 1) The presynaptic neuron releases neurotransmitter, but no receptors are present (postsynaptically silent synapse). 2) Alternatively, presynaptic release is turned off (mute synapses). Finally, 3) the synapse is anatomically pruned and thus absent altogether (although it could be recruited again [60]). The first and second would presumably allow the system to rapidly re-recruit the synapse, while the third option not only saves energy, but also reduces anatomical wiring length and volume.

It is worthwhile to ask if our model is consistent with neuroscience data. Naively, one might think that imbalance would predict that LTD would be stronger than LTP, which would contradict typical experimental findings. However, for sparse patterns LTD has to be weakened to prevent saturation, so that the imbalance condition becomes *f* ⋅ LTP < (1−*f*) ⋅ LTD. It is unclear whether this condition is fulfilled in biology. Next, one could expect that the theory would predict a net decrease of synaptic strength during learning. However, this is not the case: after all, in the simulations all weights are zero initially, so that synaptic weights can only grow during learning. The reason for this apparent paradox is that learning is gated, unlike unsupervised learning, so the number of LTP and LTD events on a synapse does not necessarily match. While our findings also hold when we start from random weights, there is no obvious initial value for biological synaptic weights.

Finally, one can compare the resulting weight distributions and the number of silent synapses to the data. An advantage of the cerebellum is that also the fraction of zero-weight synapses is known, which is not the case for other brain regions. The weight distribution in the cerebellum matches theory very well when the capacity of a two-class perceptron is maximised in the presence of noise. The fraction of silent synapses exhibits a strong dependence on the required noise tolerance; it is significantly decreased in the low noise limit [28]. Our current model finds a similar distribution from a very different objective function, namely minimising the energy of a one-class perceptron. Which of these two is the appropriate objective for the cerebellum or other brain regions remains a question for future research.

## Methods

### Criteria for optimising information

Provided that the memory problem is realisable, perceptron learning ensures that each of the *K* patterns leads to postsynaptic firing activity (*h* ≥ 0), i.e., the FN error probability is zero, *p*_10_ = 0. In this case the information increases as the FP error probability *p*_01_ decreases (see main text, Eq 7). With the additional assumption that the lures are uncorrelated to the learned patterns, it can be shown that our perceptron learning rule leads to a decrease of the FP error.

To see why, we write *p*_01_ as a function of the learned synaptic weights. As the lure patterns are uncorrelated to the learned ones, each input *x*_*i*_ will be uncorrelated to its corresponding weight *w*_*i*_. The total synaptic current is given by a sum of many terms. Assuming that there are sufficient non-zero weights, the probability distribution *p*(*h*_*l*_) of the net input *h*_*l*_ in response to a lure is Gaussian, $h_{l}∼𝓝(\langle h_{l}\rangle ,\langle \delta h_{l}^{2}\rangle )$. Under this approximation,

$$\begin{array}{c} p_{01}\approx \int_{0}^{\infty }dh_{l}\;p\left(h_{l}\right)=\frac{1}{2}\mathrm{erfc}(-\frac{\left\langle h_{l}\right\rangle }{\sqrt{2\langle \delta h_{l}^{2}\rangle }}), \end{array}$$ (12)

where $\text{erfc}(x)=\frac{2}{\sqrt{\pi }}\int_{x}^{\infty }e^{-t^{2}}\text{d}t$ is the complementary error function. The approximation improves as *N* → ∞, as the fraction of non-zero synapses *F* remains finite irrespective of the imbalance λ (for *λ* ≤ *λ*_max_) and as long as the memory load *α* does not vanish [10].

**Fig 8:**
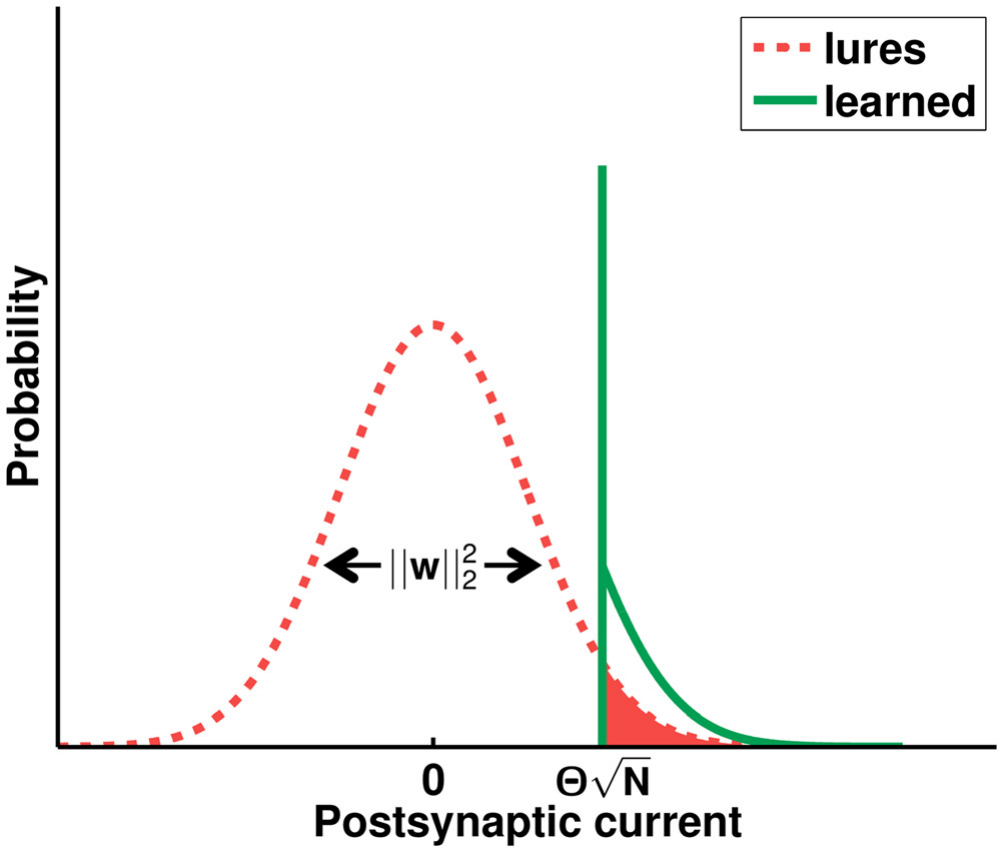
Schematic illustration of the postsynaptic current distributions. In the large *N* limit, the postsynaptic current elicited by lures (dashed line) is well described by a zero-mean Gaussian, whose variance $\left\langle \delta h_{l}^{2}\right\rangle$ is determined by the squared Euclidean norm $||\text{w}|{|}_{2}^{2}$ of the weight vector. Assuming that the learning dynamics converged, the postsynaptic current distribution provided that the input pattern is a learned one (solid line) is characteristic of perceptron learning: a significant number of patterns lie on the decision boundary and thus provoke a current that is exactly at the firing threshold, while the remaining ones generate super-threshold Gaussian tail currents [28]. The integral of the shaded area gives the FP probability *p*_01_, which depends on the variance of the lure current distribution.

As the inputs are in zero-mean bipolar form, ⟨*x*⟩ = 0, ⟨*x* ^2^⟩ = 1. The mean current elicited by lures is just $\langle h_{l}\rangle =N\langle x\rangle \langle w\rangle -\theta \sqrt{N}=-\theta \sqrt{N}$, independent of the weights. The variance in the current

$$\begin{array}{c} \left\langle \delta h_{l}^{2}\right\rangle =\left\langle {\left(\delta \left(h_{l}+\theta \sqrt{N}\right)\right)}^{2}\right\rangle =N(\left\langle x^{2}\right\rangle \left\langle w^{2}\right\rangle -{\left(\langle x\rangle \langle w\rangle \right)}^{2})=N\left\langle w^{2}\right\rangle \end{array}$$ (13)

is proportional to the second raw moment $\langle w^{2}\rangle =\int_{0}^{\infty }\text{d}w\;p(w)w^{2}$ of the weight distribution. For a particular realisation of the system one has $N\langle w^{2}\rangle =||\text{w}|{|}_{2}^{2}$, the squared Euclidean norm of the synaptic weight vector. This is illustrated in Fig 8. The information of the system is thus given by the Euclidean norm of the weight vector alone. This is true for the learned-vs-lure discrimination task as long as the Gaussianity of the lure current *h*_*l*_ holds, irrespective of the particular learning rule that is employed. For instance, *p*_01_ takes the same form for postsynaptic-independent learning [19] or for rate-coded inputs that are learned via the offline pseudo-inverse rule [22].

Thus, the perceptron with the most information satisfies the firing condition *h* ≥ 0 for every learned pattern, but has a minimal Euclidean length weight vector. This coincides exactly with the traditional perceptron that is optimal with respect to the maximal-stability criterion [39], that prescribes the weight configuration with largest stability $\Delta \equiv \theta \sqrt{N}/||\text{w}|{|}_{2}$. This is a widely used principle that enlarges the basins of attraction in recurrent networks and improves the ability to generalise in classifiers [39, 61]. Notice that for a fixed threshold, increasing Δ can only increase information, as it is inversely proportional to the Euclidean weight vector length. Information maximisation thus reveals an interesting close link between recognition memory and the more usual two-class learning problems.

Furthermore, at least for random patterns, we can expect the perceptron learning rule to perform well. Below the critical load *α*_max_ the algorithm is known to converge to solutions with large Δ [62]. In other words, although the learning dynamics is not guaranteed to maximise information, it should achieve high *C* in the recognition task. As shown in the main text, Fig 2, the improvement indeed is minimal when the full quadratic program is actually solved.

The crucial condition that must be observed to achieve good performance is that the firing threshold *θ* should be large. Here *θ* plays the role of an indirect (unnormalised) stability parameter. It can be shown [39] that raising *θ* will indirectly lead to solutions with larger Δ. Lower bounds on how close the learning rule gets to maximal stability with a certain setting of *θ* and *a*, *b* can be extracted from the perceptron convergence proof [39].

Note that the above reasoning requires zero-mean inputs and balanced plasticity. For 0 or 1 inputs, the distribution of the unthresholded output *h*_*l*_ that is obtained in response to lures is still well characterised by a Gaussian, as an uncorrelated input pattern gives a sum over on average *fN* randomly selected weights. The expressions for the mean ⟨*h*_*l*_⟩ and the variance $\left\langle \delta h_{l}^{2}\right\rangle$ now include terms that depend on first- and second-order moments of the synaptic weight distribution. For a particular realisation of the random system the mean is $\langle h_{l}\rangle =fN\langle w\rangle -\theta \sqrt{N}=f|\text{w}|-\theta \sqrt{N}$, and the variance $\langle \delta h_{l}^{2}\rangle =N\left(f\langle w^{2}\rangle \right)-f^{2}{\langle w\rangle }^{2}=f\|w{\|}_{2}^{2}-f^{2}N^{-1}|w{|}^{2}$. Thus, when the inputs are in 0 or 1 form, the information per synapse *C* is no longer a simple function of the squared Euclidean norm as before. The output error probability *p*_01_, and therefore the information, is affected by the coding level *f* and the linear norm ∣**w**∣ as well.

### Imbalanced plasticity affects convergence of the learning dynamics

To gain further insight on the effects of allowing a depression-potentiation imbalance, we prove the convergence of perceptron learning rule Eq 3 for non-zero *λ*, a variation of the detailed proof given by [29]. Besides the inclusion of the parameter *λ*, differences arise because our inputs are in bipolar form and because all patterns should elicit a high output.

We study a problem that can provably be solved in a finite number of learning steps by balanced postsynaptic-dependent learning (*λ* = 0). Therefore we can assume the existence of a weight configuration w* that solves the recognition task

$$\begin{array}{c} \sum_{i=1}^{N}w_{i}^{*}x_{i}^{k}-\left(\theta +\kappa \right)\sqrt{N}\geq 0,\;k=1,\ldots ,K, \end{array}$$ (14)

while simultaneously satisfying the *N* non-negativity constraints $w_{i}^{*}\geq 0$, *i* = 1, …, *N*. The variable *κ* ≥ 0 relates the threshold $(\theta +\kappa )\sqrt{N}$ of the solution to the threshold $\theta \sqrt{N}$ that is used in the learning algorithm.

We assume that initially all synapses are silent, i.e., we start from the *tabula rasa* condition *w*_*i*_ = 0, *i* = 1, …, *N*. Learning proceeds by presenting patterns in random order. Since plasticity only occurs when the postsynaptic current $h=\sum_{i=1}^{N}w_{i}x_{i}-\theta \sqrt{N}$ is not large enough to activate the perceptron, we index time with *m* = 1, …, *M*, *m* being incremented only when *h* < 0. Whenever each synapse *w*_*i*_ changes, it does so according to the update, Eq 3

$$\begin{array}{c} \Delta w_{i}\left(m\right)=\max\left\{-w_{i}\left(m\right),\epsilon \eta_{i}\left(m\right)\right\}, \end{array}$$ (15)

where *η*_*i*_(*m*) = *x*_*i*_(*m*)−*λ* is the weight update before rectification and **x**(*m*) ∈ {**x** ^1^, …, **x** ^*K*^} is the pattern that led to the update at time *m*.

The analysis is carried out by tracking the quantity

$$\begin{array}{c} a\left(m\right)=\frac{w^{*}\cdot w\left(m\right)}{\left|\right|w^{*}{\left|\right|}_{2}{\left||w\left(m\right)|\right|}_{2}} \end{array}$$ (16)

over time. If we find that after a finite number of updates *a*(*m*) would become larger than one, then the learning process is convergent, as the Cauchy-Schwarz inequality implies that *a*(*m*) ≤ 1. To monitor the time evolution of *a*(*m*) we bound the scalar product w* ⋅ **w**(*m*) from below and the norm ∣∣**w**(*m*)∣∣_2_ from above.

After one update, the change Δ(w* ⋅ **w**(*m*)) ≡ w* ⋅ **w**(*m*+1)−w* ⋅ **w**(*m*) in the scalar product is

$$\begin{array}{ccc} \Delta (w^{*}\cdot w\left(m\right)) & = & w^{*}\cdot \Delta w\left(m\right)\\ & = & \epsilon w^{*}\cdot \eta \left(m\right)+\sum_{i\in B(m)}w_{i}^{*}\left(\epsilon +\epsilon \lambda -w_{i}\left(m\right)\right)\\ & = & \epsilon w^{*}\cdot x\left(m\right)-\epsilon \lambda \left|w^{*}\right|+\sum_{i\in B(m)}w_{i}^{*}\left(\epsilon +\epsilon \lambda -w_{i}\left(m\right)\right)\\ & > & \epsilon \theta \sqrt{N}+\epsilon \kappa \sqrt{N}-\epsilon \lambda \left|w^{*}\right|+\sum_{i\in B(m)}w_{i}^{*}\left(\epsilon +\epsilon \lambda -w_{i}\left(m\right)\right), \end{array}$$ (17)

where *B*(*m*) = {*i*: *w*_*i*_(*m*) < *ε*+*ελ* ∧ *x*_*i*_(*m*) = −1, *i* = 1, …, *N*} is the set of all synapses that are set to zero due to the lower bound. Note that the lower bound can only be triggered by depression, which in turn can only occur for low inputs. The inequality is obtained by plugging in the definition Eq (14) of w*.

A bound on the scalar product w* ⋅ **w**(*m*) itself after *m* such updates can then be obtained by iteratively applying Eq (17):

$$\begin{array}{c} w^{*}\cdot w\left(m\right)>\epsilon m\sqrt{N}(\theta +\kappa -\frac{\lambda }{\sqrt{N}}\left|w^{*}\right|)+\sum_{l=1}^{m}\sum_{i\in B(l)}w_{i}^{*}\left(\epsilon +\epsilon \lambda -w_{i}\left(l\right)\right). \end{array}$$ (18)

Meanwhile, the change $\Delta ||\text{w}(m)|{|}_{2}^{2}\equiv ||\text{w}(m+1)|{|}_{2}^{2}-||\text{w}(m)|{|}_{2}^{2}$ in the squared norm of **w**(*m*) after one step can be obtained by expanding the square $||\text{w}(m+1)|{|}_{2}^{2}=||\text{w}(m)+\Delta \text{w}(m)|{|}_{2}^{2}$, so that

$$\begin{array}{c} {\Delta ||w\left(m\right)||}_{2}^{2}=2w\left(m\right)\cdot \Delta w\left(m\right)+{\left||\Delta w\left(m\right)|\right|}_{2}^{2}. \end{array}$$ (19)

We have Δ*w*_*i*_(*m*) ∈ {*εη*_*i*_(*m*), −*w*_*i*_(*m*)}, with *w*_*i*_(*m*) < *ε*+*ελ*, as Δ*w*_*i*_(*m*) = −*w*_*i*_(*m*) only for *i* ∈ *B*(*m*). Thus, the squared norm of the update is dominated by the terms that come from low inputs at synapses that do not cross the lower bound. This gives the inequality

$$\begin{array}{c} {\left||\Delta w\left(m\right)|\right|}_{2}^{2}<\epsilon^{2}N\left(1+2\lambda q+\lambda^{2}q\right), \end{array}$$ (20)

where $q\equiv \max_{k}1/N\sum_{i=1}^{N}\delta_{x_{i}^{k},-1}$ denotes the maximum fraction of low inputs observed across the *K* patterns.

The scalar product is expanded as before:

$$\begin{array}{ccc} w(m)\cdot \Delta w(m) & = & \epsilon w\left(m\right)\cdot \eta^{m}+\sum_{i\in B(m)}w_{i}\left(m\right)\left(\epsilon +\epsilon \lambda -w_{i}\left(m\right)\right)\\ & = & \epsilon w\left(m\right)\cdot x\left(m\right)-\epsilon \lambda |w\left(m\right)|+\sum_{i\in B(m)}w_{i}\left(m\right)\left(\epsilon +\epsilon \lambda -w_{i}\left(m\right)\right)\\ & < & \epsilon w\left(m\right)\cdot x\left(m\right)+\sum_{i\in B(m)}w_{i}\left(m\right)\left(\epsilon +\epsilon \lambda -w_{i}\left(m\right)\right). \end{array}$$ (21)

Note that the update condition *h* < 0 is always satisfied at time *m*, so that $\varepsilon \text{w}(m)\cdot \text{x}(m)<\varepsilon \theta \sqrt{N}$. Together with the bound Eq (20), iterating over Eq (19) gives

$$\begin{array}{c} {\left||w\left(m\right)|\right|}_{2}^{2}<\epsilon mN(\frac{2\theta }{\sqrt{N}}+\epsilon \left(1+2\lambda q+\lambda^{2}q\right))+2\sum_{l=1}^{m}\sum_{i\in B(l)}w_{i}\left(l\right)\left(\epsilon +\epsilon \lambda -w_{i}\left(l\right)\right) \end{array}$$ (22)

$$\begin{array}{c} <\epsilon mN(\frac{2\theta }{\sqrt{N}}+\epsilon \left(1+2\lambda q+\lambda^{2}q\right)+2q\epsilon {\left(1+\lambda \right)}^{2}). \end{array}$$ (23)

The last inequality is obtained by noticing that *w*_*i*_(*l*) < *ε*+*ελ* inside the sum over *l*; the factor *q* arises from the iteration over the *N* synapses, conditioning on the low inputs. The bound Eq (23) implies that as learning proceeds ∣∣**w**(*m*)∣∣_2_ cannot grow faster than $\sqrt{m}$.

From Eq (22) we collect

$$\begin{array}{c} \epsilon m\theta \sqrt{N}>-\frac{1}{2}\epsilon^{2}mN\left(1+2\lambda q+\lambda^{2}q\right)-\sum_{l=1}^{m}\sum_{i\in B(l)}w_{i}\left(l\right)\left(\epsilon +\epsilon \lambda -w_{i}\left(l\right)\right). \end{array}$$ (24)

Turning back to Eq (18) and using the previous result Eq (24) yields

$$\begin{array}{ccc} w^{*}\cdot w & > & \epsilon mN(\frac{\kappa }{\sqrt{N}}-\frac{1}{2}\epsilon \left(1+2\lambda q+\lambda^{2}q\right)-\frac{\lambda }{N}\left|w^{*}\right|)\\ & & \;+\sum_{l=1}^{m}\sum_{i\in B(l)}\left(w_{i}^{*}-w_{i}\left(l\right)\right)\left(\epsilon +\epsilon \lambda -w_{i}\left(l\right)\right)\\ & > & \epsilon mN(\frac{\kappa }{\sqrt{N}}-\frac{1}{2}\epsilon \left(1+2\lambda q+\lambda^{2}q\right)-\frac{\lambda }{N}\left|w^{*}\right|-q\epsilon {\left(1+\lambda \right)}^{2}). \end{array}$$ (25)

The last inequality stems from *w*_*i*_(*l*) < *ε*+*ελ*. The first bracketed factor is always larger than −(*ε*+*ελ*), while the second one is bounded from above by *ε*+*ελ*. Iterating over the constrained sum introduces the factor *Nq* as before.

We now have a bound for the cosine *a*(*m*). Substituting in Eqs (23) and (25) gives

$$a(m)>\frac{\sqrt{\epsilon mN}\left[\kappa N^{-1/2}-\lambda N^{-1}|w^{*}|-\frac{1}{2}\epsilon (1+2\lambda q+\lambda^{2}q)-q\epsilon {\left(1+\lambda \right)}^{2}\right]}{||w^{*}|{|}_{2}\sqrt{2\theta N^{-1/2}+\epsilon (1+2\lambda q+\lambda^{2}q)+2q\epsilon {\left(1+\lambda \right)}^{2}}}.$$ (26)

Note that while the neural parameters {*ε*, *θ*, *λ*} can be set at will, for a certain task the solution margin *κ* and the norms are constrained by the existence of a vector w* that can satisfy the learning conditions. Thus, they cannot be varied arbitrarily. In fact, if one keeps ∣∣w*∣∣_2_ fixed, it will only be possible to increase *κ* up to a certain point, where we will have found the maximally-stable configuration. Similarly, the linear norm ∣w*∣ will have a minimum value. Furthermore, in general it is not possible to achieve simultaneously minimal ∣w*∣ and maximal *κ* with a single configuration.

From Eq (26) a number of conclusions can be drawn. The straightforward condition for convergence is to check whether that bound becomes larger than one. Another way to show that the learning algorithm stops is to check if *a*(*m*) is a monotonically increasing function of *m*. When *λ* = 0, the process is convergent, as long as $\varepsilon \leq 2\kappa /[\sqrt{N}(1+2q)]$. For *λ* > 0, the crucial observation is that we can only show that learning converges if *κ* can be raised so as to compensate for the negative terms in the numerator.

Thus, as expected, we find that the imbalance *λ* is related to the linear norm of the solution vector (one can increase *λ* as ∣w*∣ can be made smaller), and to the occurrence of depression events (through *q*). But more importantly, *λ*_max_ writes directly as a function of *κ* as well, which here sets the task difficulty, since the maximal value for *κ* shrinks as the memory load *α* increases. What is more, the minimum of ∣w*∣ depends itself on *α*. This theoretical prediction is confirmed by our numerical work. As *α* increases, the achievable imbalance *λ*_max_ becomes closer to zero, and the fraction of silent synapses approaches that which is obtained with balanced (*λ* = 0) learning, cf. Fig 2C.

### Generating correlated patterns

We generate correlated patterns following previous work in recognition memory [21]. In the first model we generate a template pattern $\hat{\text{x}}$ with each input ${\hat{x}}_{i}$ being set high (+1) or low (-1) independently and with equal probability 1/2. To maintain balance we also use its negative, $-\text{x}-\hat{\text{x}}$, as a template.

The *K* patterns the neuron should learn are generated conditioned on either template, such that $\text{P}(x_{i}^{k}={\hat{x}}_{i})=\frac{1+g}{2}$. Lure patterns follow the statistics of the learned patterns and are produced from the same templates. The parameter *g* controls the level of input correlations; with the choice *g* = 0 the original statistics are recovered, while at *g* = 1 the recognition task is impossible, as all patterns are perfect copies or reversals of one another.

In the second model patterns generated according to the process described above, but only using a single template. This procedure introduces inter-pattern correlations at the same presynaptic site *x*_*i*_, as the arriving patterns become more similar to one another. It also leads to heterogeneous mean activity levels across neurons; although the mean number of active presynaptic neurons per pattern remains 1/2, increasing *g* leads to a bimodal presynaptic firing distribution. For *g* > 0, neurons that are active in the template fire more often and, conversely, the remaining neurons fire less frequently.

### Computer simulations

All our computer simulations were implemented on Matlab R2013a (MathWorks) and were performed on a standard desktop computer. We simulated a single postsynaptic neuron that was driven by *N* = 1000 presynaptic random inputs. We varied the memory load parameter within the range *α* ∈ [0.1,0.8] to avoid both the appearance of unsolvable problem instances and excessive simulation time. We chose a small learning rate *ε* = 1/*N* and a sufficiently large firing threshold at $\sqrt{N}$ (i.e., *θ* = 1) except when otherwise noted. The threshold was set so that typically no increase in information could be obtained by raising it further. In the figures we included second-degree polynomial fits to average values.

The online perceptron learning rule was iterated until all patterns were learned. To obtain the maximally-imbalanced solution (*λ* = *λ*_max_) we minimised the linear norm ∣**w**∣ using a linear programming algorithm [38], subject to the set of inequality constraints that ensured that every pattern would lead the neuron to fire. Specifically, using Matlab’s interior-point solver, available via the linprog command (Optimization Toolbox), we minimised ∣**w**∣ subject to *N* non-negativity constraints *w*_*i*_ ≥ 0 and *K* linear pattern imprinting constraints specified in matrix form as ${\text{X}}^{⊤}\text{w}\geq \theta \sqrt{N}\text{1}$, where **X** ^⊤^ is the *K* × *N* design matrix whose rows are the positive examples.

For the balanced case, the maximum-information weight configurations were obtained using the Krauth-Mézard min-over algorithm [39], followed by rectification after each learning step in order to enforce the non-negativity synaptic constraints. This is a batch learning algorithm that employs the balanced rule (Eq 3, *λ* = 0). At each step the pattern ${\text{x}}^{k_{\text{min}}}$ with lowest stability, $k_{\text{min}}=\arg\min_{k=1}^{K}\sum_{i=1}^{N}w_{i}x_{i}^{k}$, is determined on the forehand. Then, only ${\text{x}}^{k_{\text{min}}}$ is learned; plasticity is silenced for all other patterns. To confirm optimality and validate our mathematical results we also resorted to an interior-point convex optimiser [38] and solved the quadratic programming problem of finding the weight vector with minimal Euclidean norm ∣∣**w||**_2_. We resorted to Matlab’s quadprog command (Optimization Toolbox) to minimise $||\text{w}|{|}_{2}^{2}$ subject to the same *N* non-negativity and the *K* pattern imprinting constraints imposed on the linear program. Up to numerical precision the obtained pattern stabilities Δ matched those given by the min-over algorithm.

To calculate the information Eq (7) we tested the neuron with a set of *K* lures generated with the same statistics as the *K* learned patterns and recorded the number of FP errors. To determine the fraction of silent synapses, one has to take care of numerical rounding errors as it might be unclear when a synapse can truly be considered zero. We removed the weakest synapses one by one while probing the neuron with a large number of lures, until a drop in information occurred. With this procedure we could distinguish the true zero-weight synapses from small ones while avoiding numerical precision issues and arbitrary threshold setting. The results did not qualitatively change if we simply counted the number of synapses below some small weight $w_{\text{zero}}\ll {\text{max}}_{i=1}^{N}w_{i}$, held constant across trials.

Since we expected self-averaging of the synaptic weights distribution from the validity of the replica trick [7], the averaged synaptic weight histograms were collected from 1000 trials. To set a common weight scale across different learning rules and input statistics, we normalised the synaptic weights so that the threshold became unity, i.e., we re-scaled the weights by a factor $w_{i}/{\text{min}}_{k=1}^{K}\sum_{i=1}^{N}x_{i}^{k}w_{i}$.
